# PROTOCOL: The association between whole‐grain dietary intake and noncommunicable diseases: A systematic review and meta‐analysis

**DOI:** 10.1002/cl2.1186

**Published:** 2021-07-26

**Authors:** Wasim A. Iqbal, Gavin B. Stewart, Abigail Smith, Linda Errington, Chris J. Seal

**Affiliations:** ^1^ Newcastle University Newcastle upon Tyne UK

## Abstract

Our primary research questions are: (1) What is the association between whole grains (WG) intake and the prevalence of NCDs (i.e., type 2 diabetes, cardiovascular disease, obesity, cancer, mortality) and their biomarkers? (2) Which biomarker(s) has/have the greatest association with WG intake when combining multiple biomarkers together in the same analysis? Our secondary research question is: (3) Are there dose–response relationships between WG intake and biomarkers and prevalence of NCDs which could help inform a universal recommendation for WG intake?

## 1 BACKGROUND

### 1.1 Description of the condition

Wheat, rye, oats, barley and other varieties of grains are some of the most important staples in the human diet. With wheat alone contributing up to 68% of world food supplies, this has led to the increasing interest in the health effects of grains, in particular whole grains (WG) (Curtis et al., 2002). WGs are defined as containing all of the anatomical components of the grain including the bran, endosperm and germ (van der Kamp et al., 2014). WGs unlike refined grains (RGs) are associated with favourable reductions in incident diabetes, cardiovascular disease (CVD), cancer and all‐cause‐mortality. Studies show both weak and strong associations but there are now a large amount of reviews (both systematic and meta‐analysis) supporting the latter (McRae Marc, 2017). Despite existing evidence, current systematic reviews and meta‐analyses fail to acknowledge the range of health associations with WG in a single paper (i.e., diabetes, cancer, mortality, CVD, obesity). This leaves the health associations with WG open to more than one interpretation which serves to confuse the scientific community and public.

Noncommunicable diseases (NCDs) such as metabolic syndrome and CVD are defined by a cluster of biomarkers associated with obesity, overweight, dyslipidemia and impaired glucose homoeostasis (Alberti et al., 2005). Biomarkers such as plasma inflammatory markers, plasma glucose homoeostasis markers and anthropological measurements have provided detailed information on the association WG intake with individuals or population at risk of certain NCDs (McRae Marc, 2017). The association of WG with conditions such as mortality and cancer are difficult to quantify physiologically and have been assessed using risk ratios and rate ratios (McRae Marc, 2017). To our knowledge there is no systematic review and meta‐analysis which has given an over‐arching summary of the association WG intake on these multiple health outcomes.

The proposed review will provide clarity on the associations between WGs and NCD and will provide data to underpin the development of robust dietary WG recommendations. The EU Healthgrain Forum (www.healthgrain.org) has proposed standardised definitions of WG and WG‐foods (van der Kamp et al., 2014; Ross Alastair et al., 2017). However, in many other continents WGs are defined differently or not defined at all. A dose–response analysis (or meta‐regression) using these standardised definitions will encourage governments to adopt these definitions and set new targets for WG intake to improve population health.

### 1.2 Description of the intervention

Most current intervention and cohort studies calculate WG intake based on all foods containing 25% or 30% WG by weight depending on the definition adopted (Liu et al., 1999, 2003; Jacobs et al., 2007). WG flours are defined widely as containing all of the anatomical components of the grain, including the bran, germ and endosperm (van der Kamp et al., 2014; Ross Alastair et al., 2017). Pseudocereals such as quinoa and amaranth, and seeds of the *Poaceae* family of grasses such as rye, oats, wheat, barley, maize and rice are all included in the WG category, while legumes and oilseeds are not (van der Kamp et al., 2014). Over the years, WG consumption has been monitored in individuals with NCDs, to assess the associations between consumption of WGs and health outcomes in a longitudinal manner. Similarly, there have been many interventions involving consumption of prescribed amounts of WG foods such as bread, cereals or snacks in healthy individuals and individuals with NCDs. Cohort studies attempt to measure total WG intake whereas intervention studies attempt to prescribe consumption of specific WG foods or a range of WG foods to mimic normal consumption, or a specific level of consumption. Participants can be of either sex depending on the disease being investigated (e.g., men for prostate cancer and women for breast cancer) but most studies include randomly selected cases and/or healthy individuals from the general population. Studies mostly compare outcome measures at high WG intakes with outcome measures at low WG intakes. The lowest intake group in the intervention and cohort studies normally consume the least WG or sometimes a placebo such as RGs; both of which are considered zero WG intake (e.g., Jacobs et al., 2007; Kirwan et al., 2016). The literature contains examples of interventions that involve modified test foods such as WGs with added fibre or added bran which should not to be included alongside WG interventions (Liatis et al., 2009). Some studies also report all grains that are not separated into RG and WGs (e.g., Deneo‐Pellegrini et al., 1999; Lewis John et al., 2009) unless the intake for only WGs can be isolated from that of RGs. Only foods with their natural proportions of bran, germ and endosperm are considered WG by widely accepted definitions of WG foods such as the HEALTHgrain forum and American Association of Cereal Chemists (AACC) (Ross Alastair et al., 2017).

### 1.3 How the intervention might work

**Figure 1:**
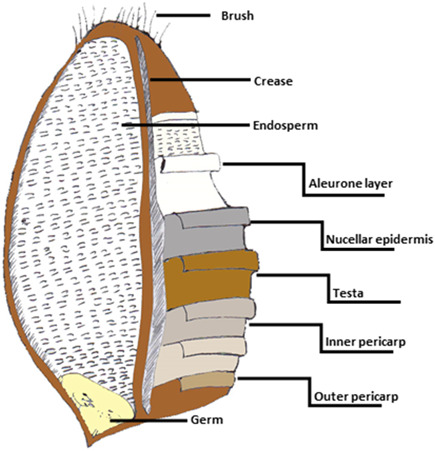
Basic structure of a WG. The bran constitutes the inner and outer pericarp, testa and nucellar epidermis. The endosperm mostly consists of starch and the aleurone layer (which is removed with bran in refinement). The germ contains the plant embryo (Evers & Millar, 2002; Slavin, 2004)

WG foods are rich in dietary fibres, vitamins, minerals, protein and phytochemicals. These vary between grain types (Jonnalagadda et al., 2011). WG flours retain the outer layers of the bran and germ which makes WG foods nutritionally more dense than RG foods (Figure 1). Each nutrient has been shown to exhibit different effects on human health.

WGs are a rich source of dietary fibres which are found mainly in the bran (Evers & Millar, 2002; Slavin, 2004). Dietary fibres from WGs are associated with greater reductions in disease risk including CVD and colorectal cancer compared with other sources of fibre such as fruit (Aune et al., 2011; Threapleton et al., 2013). The ability of WG to promote satiety, laxation, commensal gut microbiome in the large intestines, reduced blood glucose and cholesterol concentrations are believed to be the reasons for the reductions in disease cases found in cohorts consuming higher levels of WG and this may be linked to their fibre content (Fuller et al., 2016; Lutsey et al., 2007; Skeie et al., 2014). Dietary fibres are chemically the most complex biomolecules in nature and each fibre behaves differently but they have all been broadly characterised by these common physiological effects (AACC, 2001). Some studies have investigated the association of cereal fibre and WG and show that when controlling for cereal fibre intake, the significant benefits of WG still remain (Barrett Eden et al., 2019). This suggested that the health benefits of WG are not just due to fibre alone.

WGs are rich in B vitamins including folate, thiamin, niacin, riboflavin and pyridoxine (Jonnalagadda et al., 2011; Piironen et al., 2008). B vitamins have important roles in metabolic pathways. For instance, reductions in homocysteine concentrations have been observed after WG consumption, a marker of CVDs (Jang et al., 2001). This may be attributed to higher intakes of folate present in WGs which is necessary for the final enzymatic reaction in methionine metabolism (Gibney et al., 2009). Vitamin E and vitamin A (tocopherol and tocotrienol) are a fat‐soluble antioxidants found in the bran and germ layers of WGs (Lampi et al., 2008). When consumed, vitamin E is incorporated into the membrane of cells where it is able to retrieve reactive oxygen species; Molecules responsible for oxidative stress from a range of NCDs (Gibney et al., 2009). Vitamin A (retinoids and carotenoids) has important roles in cell gene expression and vision (Gibney et al., 2009). It has also been shown in animal models that high vitamin A have antiobesity effects (Berry Daniel & Noy, 2009; Felipe et al., 2003). However, most of these studies used excess vitamin A doses so it is not known if the levels of vitamin A consumed from WG are able to give similar effects.

Phytochemicals are natural components of WGs which form part of the plants natural defence such as phytosterols and phenolic acids. Phytosterols are well documented for their ability to reduce serum cholesterol concentrations through inhibition of cholesterol absorption in the small intestine (Nurmi et al., 2008; Rebello Candida et al., 2014). Phenolic acids such as ferulic acid are also well known for their roles in regulating cholesterol concentrations and their aromatic ring with more than one hydroxyl group means that they can also act as antioxidants (Borneo & Leon, 2012; Li et al., 2008; Rebello Candida et al., 2014). Therefore, phytochemicals, similar to vitamin A, E and dietary fibres, may also be responsible for the favourable associations between WG intake and disease risk (Kaur & Myrie Semone, 2020; Zhu & Sang, 2017).

Despite these associations, some WG interventions have not presented compelling evidence for associations between WG intake and various biomarkers of NCDs. For instance, an intervention on body weight and inflammation did not show an effect on C‐reactive protein (CRP; a marker of inflammation) but several observational studies have shown that WG consumption leads to a decrease in body weight (Gaskins et al., 2010; Lutsey et al., 2007). The same scenario can be applied to WG interventions on CVD, hypertension and diabetes which are associated with similar biomarkers. Observational studies involve large sample sizes and longer durations but intervention studies have much smaller sample sizes and are of relatively short duration which means that the association of WG on risk biomarkers may not be detected. For example, an oatmeal intervention did not reduce fasting glucose concentrations after a 4 weeks of intervention but after a 12‐week intervention with WG wheat and oat products a reduction in fasting glucose was found (Geliebter et al., 2014; Tighe et al., 2010).

Study locations and WG definition have also been suggested as reasons for the variations in the reported health associations of WGs. This is clearly reflected in a number of large studies conducted in European countries such as the Scandinavian HELGA study where the reference category consumed considerably more WG than studies based in the USA (Fung et al., 2010; Kyrø et al., 2013). Furthermore, a study examining body weight changes in a cohort of US men showed that body weight outcomes were different depending on the definition of WG adopted (Koh‐Banerjee et al., 2004). WG when defined using the AACC definition, which requires a WG product to have at least 25% WG on a dry weight basis appeared to have a greater association than when WG was defined using the Food and Drug Administration (FDA) definition with a greater dry weight threshold (Koh‐Banerjee et al., 2004). This supports the need for a universally accepted definition of WG foods to be applied in research studies.

### 1.4 Why it is important to do this review

To our knowledge, there are only a few systematic reviews which have discussed the association WG intake on a limited range of NCDs in a single paper. Aune et al. (2016) reported the association WG on incidence of CVD, cancer or all‐cause‐mortality. Zhang et al. (2020) reported the association WG on digestive tract cancers. Ye et al. (2012) explored the association of WG on incidence of type 2 diabetes, CVD and weight gain and recently Reynolds Andrew et al. (2020) reported the association WG with diabetes risk. Ye et al. (2012) examined biomarkers of NCDs such as fasting glucose concentrations but these were shown as weighted mean differences (MDs) which appear to show that WGs have significant effects. Pol et al. (2013), Hollænder Pernille et al. (2015), Harland Janice and Garton Lynne (2008), Kelly Sarah et al., (2017), Wang et al. (2020), Sadeghi et al. (2020), and Barrett Eden et al. (2019) are the only reviews which have examined the association WG intake on biomarkers of body weight and CVD. However, these reviews did not examine evidence from observational studies and controlled trials looking at a wider range of diseases and biomarkers together. Therefore, there is the need for the formation of a high quality systematic review and comprehensive meta‐analyses which (a) adheres to the Methodological Expectations of the Campbell Collaboration Intervention Reviews (MECCIR) and (b) assesses the association WG intake on multiple health outcomes from both observational studies and controlled trials.

## 2 OBJECTIVES

Our primary research questions are:

1. What is the association between WG intake and the prevalence of NCDs (i.e., type 2 diabetes, CVD, obesity, cancer, mortality) and their biomarkers?
2. Which biomarker(s) has/have the greatest association with WG intake when combining multiple biomarkers together in the same analysis?
  Our secondary research question is:
3. Are there dose–response relationships between WG intake and biomarkers and prevalence of NCDs which could help inform a universal recommendation for WG intake?

## 3 METHODS

### 3.1 Criteria for considering studies for this review

#### 3.1.1 Types of studies

Observational studies on a mixed WG diet (e.g., a mixture of WG products such as WG bread or WG biscuits) or WG specific foods (e.g., WG rye bread) including cross‐sectional, case‐control and cohort studies will be included. Intervention studies such as randomised controlled trials (RCTs) on WG specific foods or mixed WG diet will also be included.

#### 3.1.2 Types of participants

Participants of either sex, all demographics and ages will be considered including those from both high and low income countries. Individuals classified as “cases” in this review are any individuals (i.e., healthy individuals, individuals predisposed to an illness or those with an illness) with high WG intake and individuals classified as “control” are any individuals with low WG intake or no WG intake.

#### 3.1.3 Types of interventions

In this review, studies looking at the association between WG intake and health outcomes will only be included if they include WGs with the bran, germ and endosperm in their natural proportions as defined by widely accepted definitions of WG foods (Ross Alastair et al., 2017). Cohort studies, cross sectional and case‐control studies tend to include total WG intake of a WG diet or total WG products consumed, and will be included. RCTs which include WG specific foods or a range of WG foods to mimic a WG diet will be included. These studies compare higher WG intake with either a low intake category or a placebo such as RGs, both of which will be considered zero WG intake (Jacobs et al., 2007; Kirwan et al., 2016). As previously mentioned, studies that involve modified test foods with added bran or fibre should not be confused with normal WG intake and will be excluded. Modified test foods will also not be considered as appropriate control group since they also have associations with health outcomes (see, Barrett Eden et al., 2019) and could increase between‐study inconsistency making it difficult to gauge the overall associations of WG with health outcomes in the present review. Some studies report WGs as “grains” when this may imply both RGs and WGs (e.g., Deneo‐Pellegrini et al., 1999; Lewis John et al., 2009). These studies will be excluded unless WG consumption can be isolated from that of RGs. Studies may report data for the sample population but based on different types of WG (e.g., oatmeal or rye). In such cases, study outcomes for the same population will be pooled to give a hypothetical WG diet group to avoid incorporating the same populations in statistical analyses more than once. Studies may express WG intake as portions, servings or grams. In this review, a portion or serving will be considered as 16 g of WG.

All follow‐up durations will be considered. Interventions on WG consumption range between 2 and 16 weeks and cohorts range 7–20+ years. To reduce variation and duplication of data analysis only the most up to date follow‐up time for specified cohort studies will be included. Intervention studies with 2–11 weeks of intervention will be separated from interventions of 12+ weeks duration.

#### 3.1.4 Types of outcome measures

##### Primary outcomes

Biomarkers and anthropological measurements will be considered as primary outcome measures for NCDs including obesity, CVD, type 2 diabetes and hypertension. These will include: serum fasting glucose; fasting insulin and measures of glucose/insulin metabolism including homoeostatic model of assessment (HOMA) and quantitative insulin sensitivity index (QUICKI); plasma total cholesterol, plasma low‐density lipoprotein (LDL)‐cholesterol and plasma high‐density lipoprotein (HDL) cholesterol; plasma triaclyglycerols; markers of inflammation including plasma CRP, plasma interleukins, plasma tumour necrosis factor and plasma a‐amyloid; plasma homocysteine; body fat mass, fat free mass, body mass index, waist circumference and systolic and diastolic blood pressure.

##### Secondary outcomes

Secondary outcomes will include measures from observational studies such as risk ratios, hazard ratios, mortality rate ratios, incident rate ratios.

### 3.2 Search methods for identification of studies

#### 3.2.1 Electronic searches

An example search strategy for MEDLINE can be found in the appendix which will be closely duplicated in three more databases; Web of Science, Scopus and CAB global health. Search terms were formulated based on population, intervention, conditions, outcomes and studies (PICOS) (Table 1). Ongoing studies will be identified using clinical trial registers including World Health Organisation's (WHO) clinical trials registry platform and Clinicaltrials.gov. In addition to peer reviewed articles, conference proceedings will be identified using Scopus. Thesis and dissertations will be identified using an online international database (https://oatd.org/).

**Table 1:** PICO

Population	Intervention	Condition	Outcomes	Studies
All regions, demographics, ethnicity and sex, including those from high income and low income countries	Mixed WG foods or WG specific foods based on the HEALTHgrain forums European definition of WGs. These include Wheat, spelt, emmer, faro, einkorn, Khorasan, durum, rice, barley, maize, corn, rye, oat, millet, Teff, Triticale, Canary, Job's tears, Fonio black fonio, Asian millet, Amaranth, Buckwheat, Quinoa	Healthy individuals or individuals with a non‐communicable disease, for example, type 2 diabetes, nonalcoholic fatty liver disease, metabolic syndrome, obesity, cardiovascular disease, coronary heart disease, ischaemic stroke and hypertension	Blood measurements, blood pressure measurements, anthropometric measurements and exposure ratios (e.g., hazard ratio, relative risk, incidence ratio, mortality ratio)	Randomised controlled trials, case‐control, cross‐sectional and cohort studies

#### 3.2.2 Searching other resources

References in the latest meta‐analyses, systematic reviews and included studies will also be searched for additional studies. Experts and authors of existing systematic reviews/meta‐analyses on WG will be contacted regarding ongoing studies and unpublished data.

### 3.3 Data collection and analysis

#### 3.3.1 Description of methods used in primary research

Observational studies such as cohort studies and cross‐sectional studies involve random sampling from a population that meet a specific eligibility criteria (Kasum et al., 2001; Steffen et al., 2003) will be considered. Cohort studies involve following individuals prospectively over a long period of time, while cross‐sectional studies involve collecting information on participants at a single point in time. The methods used in both types of studies are similar. Cohort studies and cross‐sectional studies mostly adopt food frequency questionnaires (FFQs) for collecting dietary intake data as they are often large studies which endure a lot of cost. FFQs provide a relatively inexpensive and fast method for collecting habitual dietary data. FFQs are normally given at the beginning of the study (baseline) and at the end of the specified follow‐up period. Other data collected during the follow‐up period may include recording of events such as death, cause of death and incidence of specified diseases. Cohort studies may assess this by medical records at baseline and at the end of follow‐up or by direct contact with participants. All cohort studies and cross‐sectional studies will most likely involve the collection of anthropometric data. Some cohort studies and cross‐sectional studies also collect blood samples such as in the MESA and Framingham cohort studies (Lutsey et al., 2007; McKeown et al., 2002).

Case‐control studies, like cohort studies and cross‐sectional studies, are observational. These studies involve collecting data from a selection of healthy individuals that do not show symptoms of the NCD (controls) and individuals that do show symptoms of the NCD (cases); the groups should otherwise be identical. For instance, a study in Switzerland examined at WG consumption of healthy individuals compared with individuals with oral, oesophagus and laryngeal cancers (Levi et al., 2000). WG case‐control studies may involve collecting dietary intake data using 24 h recalls by interview, food diaries or FFQs. They may also involve the collection of blood samples and anthropometric measurements.

RCTs involve exposing participants to a particular WG treatment (e.g., wholemeal rye bread) or group of foods (e.g., WG snacks) for a specified duration. RCTs may have a two‐arm parallel design which involves random allocation of participants into two groups with one group receiving the intervention food (e.g., WG bread) and the other either no treatment or the same food not containing WG (e.g., RG bread). For example, the WHOLEheart study included a control group that maintained their normal dietary intake and an intervention group which was asked to consume 60–70 g WG food per day (Brownlee et al., 2010). RCTs may also adopt a crossover design which involves all participants receiving all treatments in random order. A wash‐out period separates the two intervention periods to prevent carryover effects for example (Kirwan et al., 2016). Ideally participants and researchers should be blinded to the treatment in RCTs. However, WG interventions may not be adequately blinded as the appearance of WG foods are often easily distinguished from RG comparators. RCTs normally involve the collection of blood samples and anthropometric data at the beginning and end of the intervention periods.

#### 3.3.2 Selection of studies

Articles will initially be screened by title and abstract. Articles that do not specifically have “WG” in the title or abstract but do have phrases such as “dietary fiber” or “cereals” will be evaluated fully. In past efforts, studies on dietary fibre and cereals have been shown to contain information on WG (e.g., Li et al., 2015). Studies which measured outcomes but not reported them will not be excluded. All studies that meet the initial screening will be organised and compiled in a Reference Database. Screening will be conducted by two authors (W. A. I. and A. S.) and any disputes will be discussed and resolved with the corresponding author (C. J. S). A flow chart will be produced to show the number of studies excluded and included.

#### 3.3.3 Data extraction and management

Studies will initially be identified based on PICOS (Table 1).

Studies that meet PICOS will be fully analysed and will be included if they have information on the following:

- Blood measurements for one or more of the following: fasting insulin, fasting glucose, cholesterol (total or LDL or HDL), triacylglycerols, HOMA, QUICKI, CRP, a‐amyloid, Interleukin subclasses, tumour necrosis factor, leptin, glycosylated haemoglobin and homocysteine.
- Anthropometric data for one or more of the following: total body weight, fat free mass, fat mass, body mass index and waist circumference, systolic blood pressure diastolic blood pressure.
- Summary measures such as relative risk ratios, hazard ratios, mortality rate ratios or incidence rate ratios.

Data will be extracted in duplicate by two authors using an Excel sheet.

#### 3.3.4 Assessment of risk of bias in included studies

The Cochrane ROBINS risk of bias tool will be used to assess all observational studies (e.g., cross‐sectional, cohorts and case‐controls). This tool has been chosen as unlike other risk of bias tools, the ROBINS tool takes into account internal validity and attempts to mimic an RCT (Sterne Jonathan et al., 2016). This allows for more rigorous evaluation of bias that can arise from potential confounding of the effect, recall bias, differential misclassification of outcome and/or intervention status, attrition bias, reporting bias and detection bias. Key bias domains for WG studies have been identified as classification of intervention and bias due to confounding of effect. With no standardised or universal definition of WG many studies classify WG in different ways. Studies classifying WGs as having bran, germ and endosperm in their natural proportions but not an exact definition will be considered moderate risk of bias and studies using the FDA, AACC‐I and the HEALTHgrain forum definitions as low risk of bias (Ross Alastair et al., 2017). A potential list of confounding domains relevant to most studies has been identified (refer to Appendix). For RCT studies, the Cochrane risk of bias tool for RCTs will be adopted (Higgins & Green, 2011). RCTs will be screened for participant selection, outcome, attrition and reporting bias accordingly.

These forms of bias may inform reasons for heterogeneity in effect sizes. However, not all study outcomes may be affected by the same bias. For example, reporting bias may be detected in interviews or FFQs but this may not be applicable for data from analysis of blood samples. Therefore, risk of bias will be considered across all outcomes included in studies for both RCTs and observational studies.

#### 3.3.5 Measures of treatment effect

All data will be collected using Microsoft Excel. For each study, outcome data will be converted into common units. A parametric bootstrapping procedure with a minimum of 10,000 iterations will be conducted in R to obtain standard errors for studies that do not report SDs, SEs, confidence intervals (CI) or interquartile ranges (IQR) using the “Simpleboot” R package. Studies that do report SE, CI or IQR will be converted into standard deviations using equations by Borenstein et al. (2009) and Wan et al. (2014). Studies that report separate means for males and females will be combined using a fixed effect model. Studies sometimes include more than one type of WG intervention group for the same population (e.g., rye vs. oat vs. wheat vs. control). In such cases, each group will be combined using a fixed effect model to give a hypothetical mixed WG group before comparison with control group. This is to ensure that populations are not included in meta‐analyses more than once. Biomarker outcomes for WG groups and control groups will be compared using MDs in meta‐analyses. To ensure ratio outcomes are closely related before being combined in the same analyses, ratio outcomes will undergo log transformation and risk ratios will be converted to rate ratios using the follow‐up length.

#### 3.3.6 Unit of analysis issues

Crossover RCT Intervention studies using the same population as both the controls and cases are mostly separated by a wash‐out period as previously described. The results at the end of the wash out period (before intervention) will be considered as the control and results at the end of each intervention period will be considered the final outcomes for each treatment. Some intervention studies also take measurements at different time points (e.g., at 2, 3 and 4 weeks of each treatment). The time points where no WG intervention was introduced will be considered as the control and the final time point, the comparator outcomes (e.g., week 4). RCTs sometimes adopt three‐arm designs or multi‐arm designs (e.g., rye vs. wheat vs. oats) where each group is considered an intervention group and no low WG or RG comparison group is available (Dinu et al., 2017; Whittaker et al., 2017). RCTs without a control group will be excluded. For observational studies which involve grouping the population by total WG intake or WG diet, with individual findings for each group, the group with the lowest intake will be used as the reference group or control and the group with the highest intake will be used as the intervention group. However, all WG intake groups can be incorporated as individual dose‐responses in dose–response analyses. No baseline values will be considered in observational studies except for case‐control and cross‐sectional studies where the intention is not to observe a long‐term cause and effect relationship.

Because of the duration of many cohort studies, results for the same populations such as the Nurses Health Study may be reported with different follow‐up periods, as previously described. If the studies based on the same outcomes (e.g., diabetes) have more than one publication, the study with the latest follow‐up time will be chosen and others excluded to avoid duplication of data.

#### 3.3.7 Dealing with missing data

Study authors will be contacted for data that may have been measured but not reported.

#### 3.3.8 Assessment of heterogeneity

Cochrane *Q* and *I* ^2^ statistics will be used to display between‐study heterogeneity and inconsistency along with its corresponding p‐value. A *p* < .05 will be considered significant inconsistency and *I* ^2^ > 50% will be considered substantial inconsistency.

#### 3.3.9 Assessment of reporting biases

Risk of bias will be accompanied by funnel plots with the trim and fill method to assess publication bias (Higgins & Green, 2011).

#### 3.3.10 Data synthesis

In statistical analyses and as previously mentioned, “cases” will be healthy individuals, individuals predisposed to an illness or those with an illness with high WG intake and “controls” will also be healthy individuals, individuals predisposed to an illness or those with an illness but with low WG intake or no WG intake. Statistical analyses will be carried out for RCTs and observational studies separately.

##### Univariate meta‐analyses

Study outcomes will be pooled in univariate meta‐analyses using the “metafor” package in the R program (http://www.metafor-project.org/) which adopts the restricted maximum likelihood estimator for computing heterogeneity by default. Only the pooled effect size for each outcome will shown in forest plots of the main text. Full forest plots featuring the pooled effect size and the contribution of each study effect size will be shown in supplementary material.

##### Multivariate meta‐analyses

Studies reporting common biomarkers will be included in a multivariate meta‐analysis using the “metafor” package in the R program. Biomarkers will be combined in multivariate meta‐analysis by converting all biomarkers to a unit less quantity (i.e., *z* scores also known as the standardised MDs). Biomarkers will be converted to their original units when shown in forest plots for easy interpretation.

##### Meta‐regression

Linear dose–response relationship between WG intake (e.g., g/day) and MDs or ratios between control and WG intake groups will be assessed using meta‐regression with a random effects model. Studies reporting WG intake in different units will be converted to g/day. As previously mentioned, a serving of WG will be considered 16 g/day which will be used to recalculate data from studies reporting servings/day to g/day. WG intakes expressed as IQR will be averaged to obtain an estimate for the median. When the highest bound is open ended it will be assumed that it is the same as the adjacent interval. When the lower bound is open ended it will be assumed that it begins from 0.

#### 3.3.11 Subgroup analysis and investigation of heterogeneity

Between‐study inconsistency will be explored by stratifying univariate meta‐analyses by medical status (i.e., healthy vs. metabolic diseases), study duration, WG definition, gender, age and study location.

Statistical analysis will be conducted by two authors (G. S. and W. I.) and the data extraction will be conducted by two authors (W. I. and A. S.) and checked by another (C. J. S.).

#### 3.3.12 Sensitivity analysis

Based on the overall bias of each study, sensitivity analyses will be conducted to assess the robustness of results with or without inclusion of studies judged to have high risk of bias. Studies that are classed as “unclear” will be considered less than “high risk” as outlined in the Cochrane handbook (Higgins & Green, 2011).

#### 3.3.13 Summary of findings and assessment of the certainty of the evidence

Qualitative information will be collected on dietary assessment method, age, sex, number of participants, duration of study, outcomes measured, WG intake, WG definition, study design, type of WG, location of study and adjustments made to outcomes. The data will be summarised in a study characteristics table.

## CONTRIBUTIONS OF AUTHORS

### Content

The content of the review was conceived by Chris J. Seal, an international expert on the health benefits of whole grain who has published several original research articles and reviews on the topic, and Wasim A. Iqbal, a research student. The structure of the review was developed with input from Gavin B. Stewart, Linda Errington, and Abigail Smith.

### Systematic review methods

Systematic review methods were designed by Gavin B. Stewart, Senior Lecturer in Evidence Synthesis. Gavin B. Stewart has an extensive publication record of meta‐analyses and systematic reviews, including publications on relevant research methods.

### Statistical analysis

Statistical methods were designed by Wasim A. Iqbal and confirmed by Gavin B. Stewart.

### Information retrieval

Search terms and the search strategy were devised by Wasim A. Iqbal, Linda Errington, and Chris J. Seal. Data will be extracted into a custom‐made Excel database by Wasim A. Iqbal and checked by Abigail Smith and Chris J. Seal. Risk of bias analysis will be conducted by Wasim A. Iqbal and checked by Abigail Smith.

## DECLARATIONS OF INTEREST

Chris J. Seal is a Board Member of the European Healthgrain Forum, Trustee and Director for the Nutrition Society, member of the Institute of Food Science and Technology (IFST) and the Association for the Study of Obesity.

Gavin B. Stewart is an Associate Editor for Peer J, Associate Editor for Research Synthesis Methods, and Co‐Chair and Senior Editor of the Campbell Collaboration.

Wasim A. Iqbal, Linda Errington, Abigail Smith have no interests to declare.

## SOURCES OF SUPPORT

### External sources

- Gilchesters Organic, UK.

## APPENDICES A

### Search strategy

Database: Ovid MEDLINE(R) <1946 to April Week 5 2020>

1. Whole Grains/
2. Edible Grain/
3. wholemeal.mp.
4. cereal.mp.
5. exp Poaceae/
6. wheat.mp.
7. spelt.mp.
8. emmer.mp.
9. faro.mp.
10. einkorn.mp.
11. khorasan.mp.
12. durum.mp.
13. rice.mp.
14. barley.mp.
15. maize.mp.
16. corn.mp.
17. rye.mp.
18. oat*.mp.
19. millet.mp.
20. Teff.mp.
21. canary.mp.
22. job's tears.mp.
23. fonio.mp.
24. buckwheat.mp.
25. Chenopodium quinoa/
26. or/1‐25
27. Noncommunicable Diseases/
28. exp Diabetes Mellitus/
29. exp Neoplasms/
30. exp Hyperglycemia/
31. exp Fatty Liver/
32. exp Obesity/
33. exp Cardiovascular Diseases/
34. exp Coronary Disease/
35. isch*emic stroke.mp.
36. exp Stroke/
37. exp Hypertension/
38. high blood pressure.mp.
39. Overweight/
40. exp Mortality/
41. Metabolic Syndrome/
42. exp Inflammation/
43. or/27‐42
44. Blood Pressure/
45. anthropometry/
46. anthropometric.mp.
47. weight.mp.
48. exp Lipids/
49. exp Cholesterol/
50. exp Glucose/
51. exp Insulin/
52. homa.mp.
53. quicki.mp.
54. exp Triglycerides/
55. C‐Reactive Protein/
56. exp Interleukins/
57. Leptin/
58. systole/
59. diastole/
60. ratio.mp.
61. risk.mp.
62. or/44‐61
63. (case adj3 control).mp.
64. (random* adj3 control*).mp.
65. cohort.mp.
66. longitudinal.mp.
67. cross sectional.mp.
68. or/63‐67 (2026164)
69. 26 and 43 and 62 and 68

### Considerations for confounding domains

Confounding domains relevant to all or most observational studies when assessing for risk of bias include:

- Hormone replacement therapy use (only females)
- Menopausal status (females)
- Alcohol consumption
- Smoking
- Family history of diseases (for studies based on diabetes, hypertension, cardiovascular disease and cancers)
- Weight (e.g., body mass index or body weight (kg))
- Socioeconomic status (eg. income, educaon or marital status)
- Fruit and vegetable intake
- Physical acvity or exercise
- Multivitamins
- Dietary fibers from sources other than whole grain foods
- Total energy intake
